# Supplementary material for: Single-Cell Approach Reveals Intercellular Heterogeneity in Phage-Producing Capacities
Source: Microbiol Spectr. 2022 Dec 21;11(1):e02663-21. doi: 10.1128/spectrum.02663-21 (PMC9927085; doi:10.1128/spectrum.02663-21)
Supplement: Supplemental file 1 — Supplemental material. Download spectrum.02663-21-s0001.pdf, PDF file, 0.3 MB [file spectrum.02663-21-s0001.pdf]

Supplementary Information

Pg. 2 – Figure S1. Chloroform treatment in 96-well plates reduces phage titer.

Pg. 3 – Figure S2. Chemically and naturally lysed cells show a similar burst size.

Pg. 4 – Cell volume measurements

Pg. 5 – Figure S3. Cell volume increases after lytic induction.

Pg. 6 – Figure S4. Burst size is positively correlated with cell volume.

Pg.7 – Single-cell burst size determined using FACSAria cell sorting

Pg. 8 – Figure S5. Burst size distribution of wild type phage λ.

Pg. 9 – Figure S6. Chemically and naturally lysed cells show a similar cell-to-cell variation (noise) in burst size (CV^2).

Pg. 10 – Fig. S7. After removing outliers, cell-to-cell variation (noise) in burst size remains constant with increasing mean burst size.

Pg. 11 – Supplementary Table 1

Pg. 12 – References

Effect of experimental conditions on phage stability

Aliquots of phage λ_{Sam7} lysate (200 µl) were dispensed into the wells of a 96-well plate to simulate the conditions used in the burst size assay (for details see Methods). The treatment involved exposing the lysate to chloroform for 20 minutes, which included 10 min of shaking at 37° C inside the plate reader followed by 10 min incubation at room temperature without shaking. A 100 µl aliquot from each well was thereafter removed for plaque assays and the estimated plaque forming units were compared to a control group that was plated without the treatment.

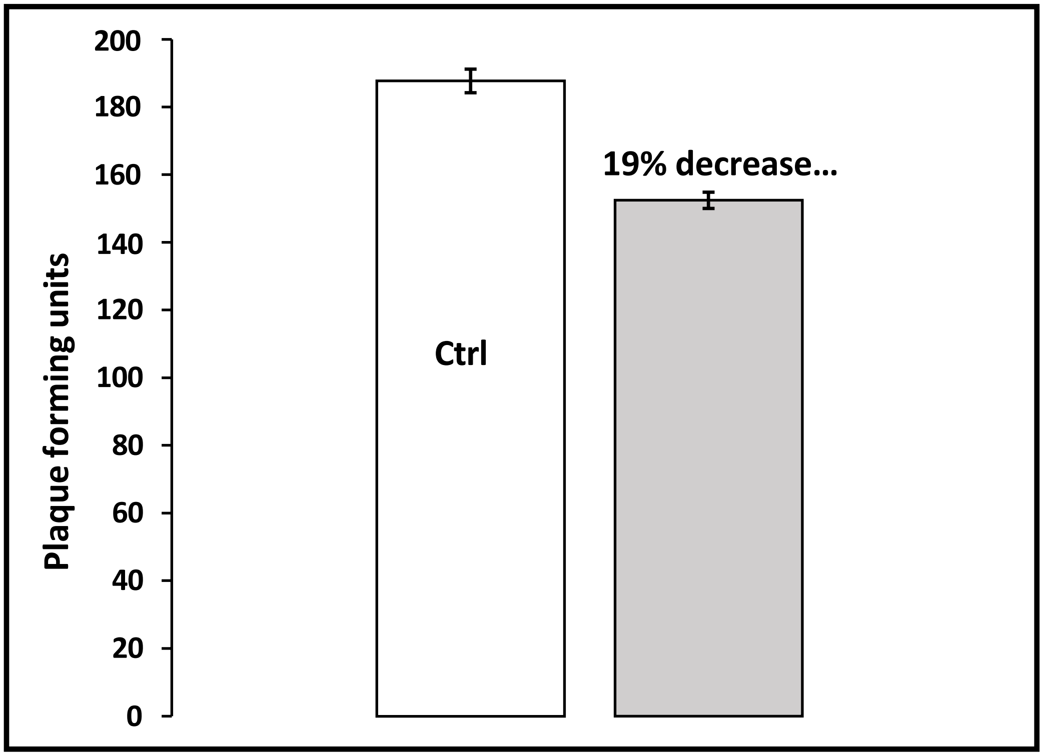

Figure S1. Chloroform treatment in 96-well plates reduces phage titer. Experimental conditions used in the burst size assay reduced phage titers by 19% when compared to a control (ctrl) group (p < 0.001; t test; n = 32). Error bars, mean ± SEM.

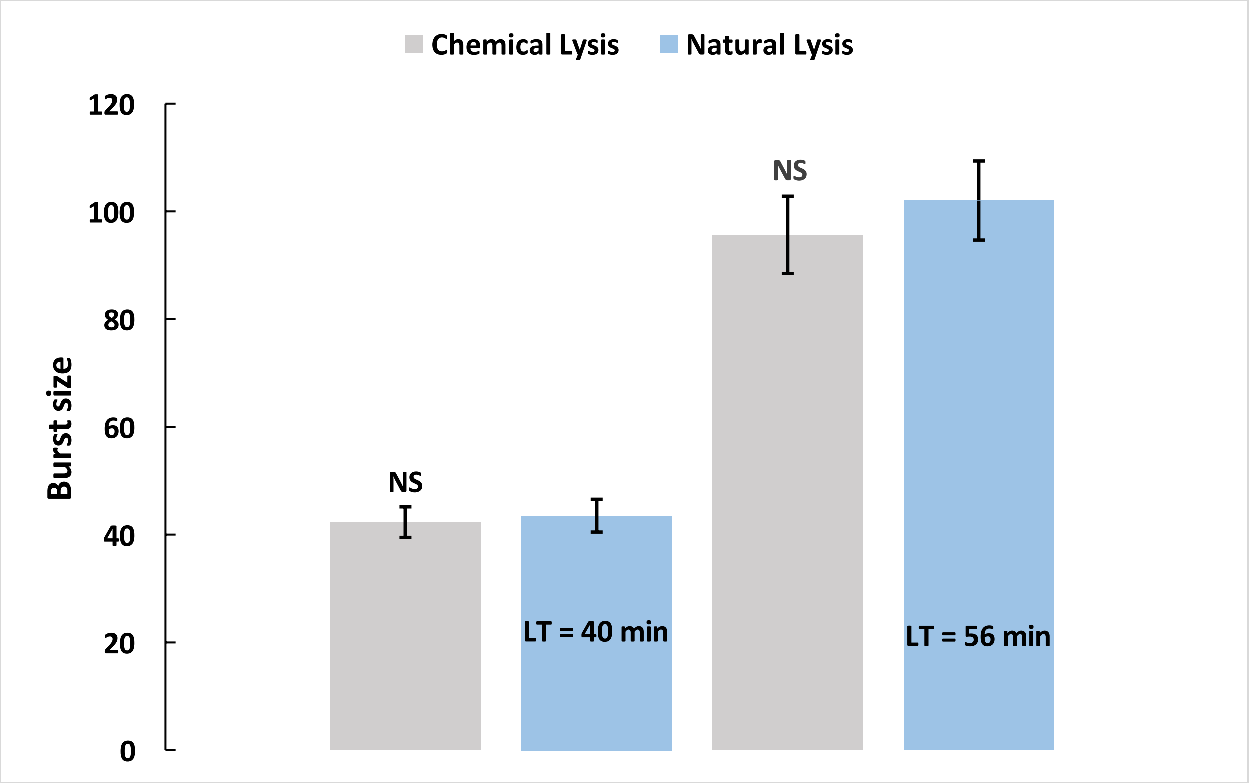

Figure S2. Chemically and naturally lysed cells show a similar burst size. An induced culture of *E. coli* lysogen with lysis-deficient λ phage was chemically lysed using chloroform to estimate burst sizes at different lysis times. These burst sizes were compared to those of naturally lysing strains with similar mean lysis times. NS, not significant (*t* test); Error bars, mean ± SEM.

Cell volume measurements

We measured cell dimensions using a simple microscopic setup described previously (Dennehy and Wang, 2011). Briefly, the lysis-deficient lysogen was grown to A_{600} = 0.3–0.4 at the permissive temperature of 30°C. The cells were then immobilized to a 22 mm glass coverslip that was pretreated with 0.01% poly-L-lysine (mol. wt. 150 K-300 K; Millipore Sigma, St. Louis, MO) for 10 min. The coverslip was assembled as a perfusion chamber (RC-21B, Warner Instruments, New Haven, CT) and attached to a heated platform (PM2; Warner Instruments, New Haven, CT). The platform was then mounted onto an inverted microscope stage (TS100, Nikon, Melville, NY). The perfusion chamber was infused with heated LB at 30°C (Inline heater: SH-27B, dual channel heating controller: TC-344B; Warner Instruments, New Haven, CT). The lytic cycle was induced by raising the chamber temperature to 42°C for 20 min. After heat shock, the temperature was maintained at 37°C. The cell growth was captured as a video using an eyepiece camera (10× MiniVID™; LW Scientific, Norcross, GA, 10 fps). Video screenshots taken at different times were used as input files for ImageJ software (Schneider, Rasband and Eliceiri, 2012), which was used to measure cell lengths and widths. A micrometer slide was used to set the scale (5.0887 pixels/µm) in ImageJ. The volume (V) of each cell was approximated as the volume of a capsule with cylindrical radius equal to the radii of the hemispheres on either ends:

$$V = \pi \cdot \frac{w^2}{4} \cdot \left[L - \frac{w}{3}\right] ,$$

where L and w are the measured length and width of a cell, respectively.

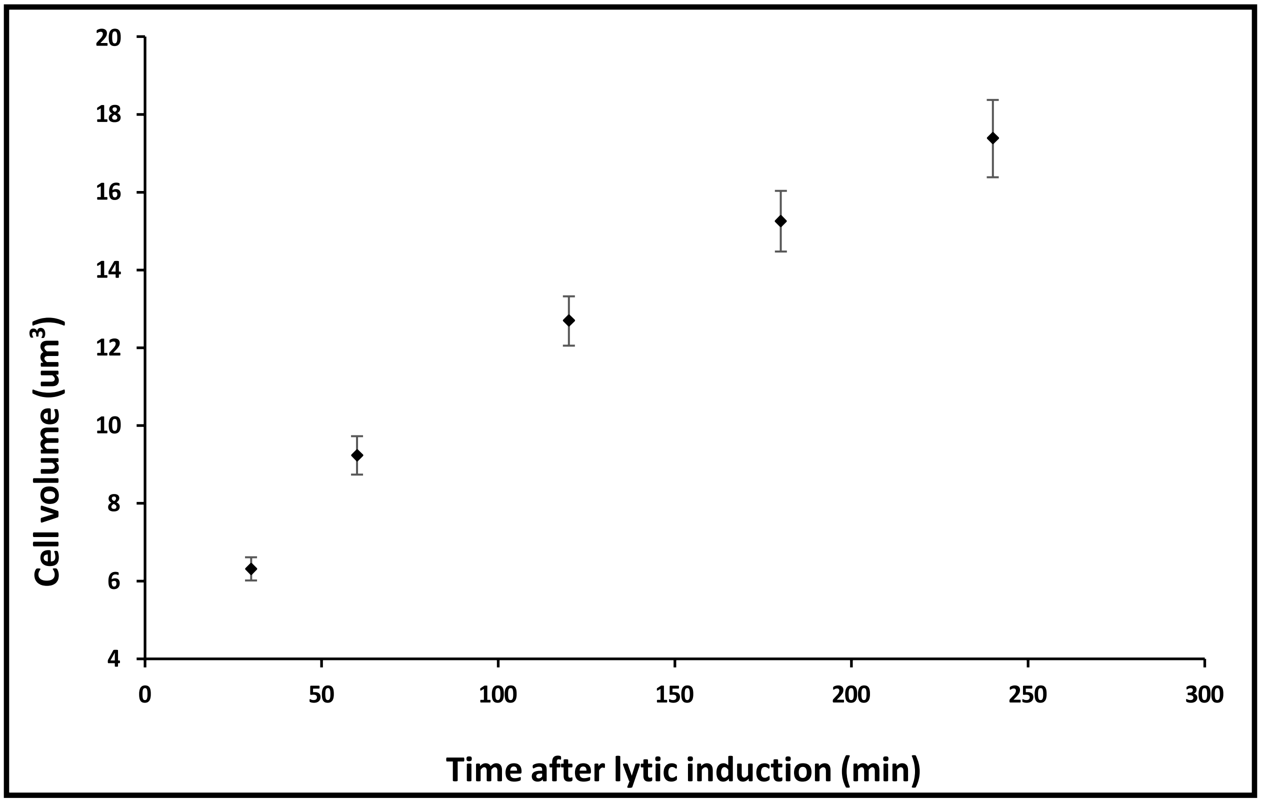

Figure S3. C**ell volume increases after lytic induction.** Lysis-deficient lysogens were heat induced to initiate the lytic cycle. Length and width of individual cells (n = 90) at different times after induction were measured to estimate total cell volume. Error bars, 95% CIs after bootstrapping (1,000 replicates).

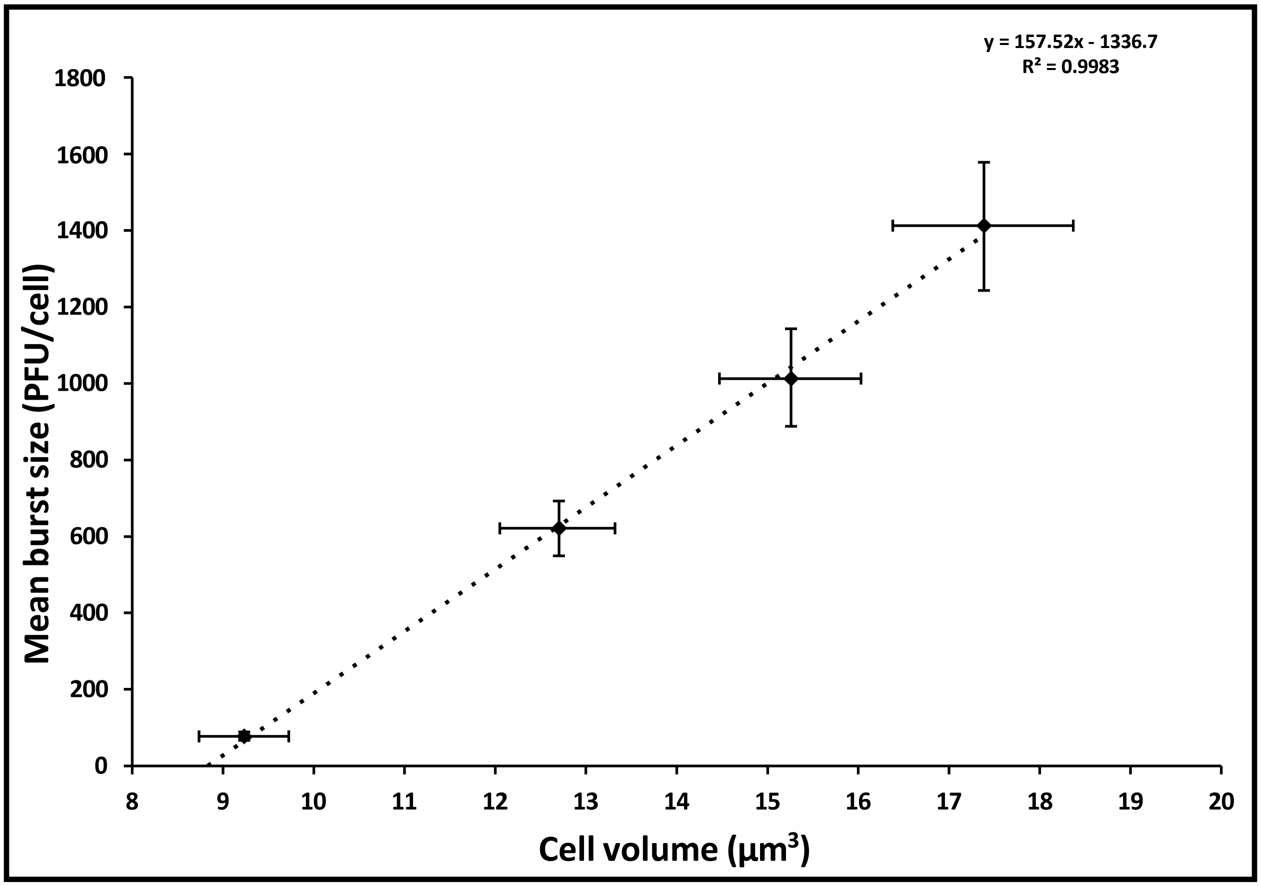

Figure S4. Burst size is positively correlated with cell volume. Burst sizes and cell volumes estimated at different time intervals after lytic induction are positively correlated. Error bars, 95% CIs after bootstrapping (1,000 replicates); dotted line, linear fit ($R^2 = 0.9983$, $p < 0.001$).

Single-cell burst size determined using FACSAria cell sorting

To prepare cells for fluorescence assisted cell sorting (FACS), single colonies of *E. coli* lysogen MC4100 (λ cI857 S105) were placed in 10 mL LB medium and cultured overnight at 30° C in a water bath shaker rotating at 250 rpm. Stationary phase cultures were spun at 3,000 rpm for 10 min, and the pellets were resuspended in 3 mL fresh LB. Subsequently, 30 µL fluorescein isothiocyanate conjugate (FITC; Virostat) was added and the cultures were returned to the water bath shaker. After 1 hr, free FITC was removed by centrifuging the cultures at 3,000 rpm for 5 min and adding 3 mL phosphate-buffered saline (PBS) to the pellet. The wash step was repeated for a total of three washes.

Cell sorting was performed using a FACSAria instrument (BD Biosciences) equipped with a 70 µm nozzle and a blue 488 nm laser. PBS was used as a sheath fluid. A preliminary analysis indicated that cells fell into two subpopulations based on the amount of laser excitation energy released and detected by photomultiplier tubes (PMT2). Since fluorescence intensity is proportional to cell size, gating was used to select cells that fell within a narrow range within the subpopulation expressing the lower amount of fluorescence intensity to minimize variation according to cell size.

This strategy was used to sort single-cells into wells of a round bottom 96-well plate (Corning) where each well contained 100 µL LB medium. The plates were placed on ice until being placed in a 42° C incubator for 15 min for induction of lysis. The plate was then incubated in a 37° C incubator for 1.5 hrs. Subsequently, 100 µL of an exponential culture (OD_{600} = 0.2) of *E. coli* MC4100 was added to each well and the cultures were allowed to rest for 15 min for phage adsorption. The entire contents of the well were then removed and added to 3 mL LB top agar, which was then poured on 35 mL LB bottom agar and incubated overnight at 37° C to visualize plaques.

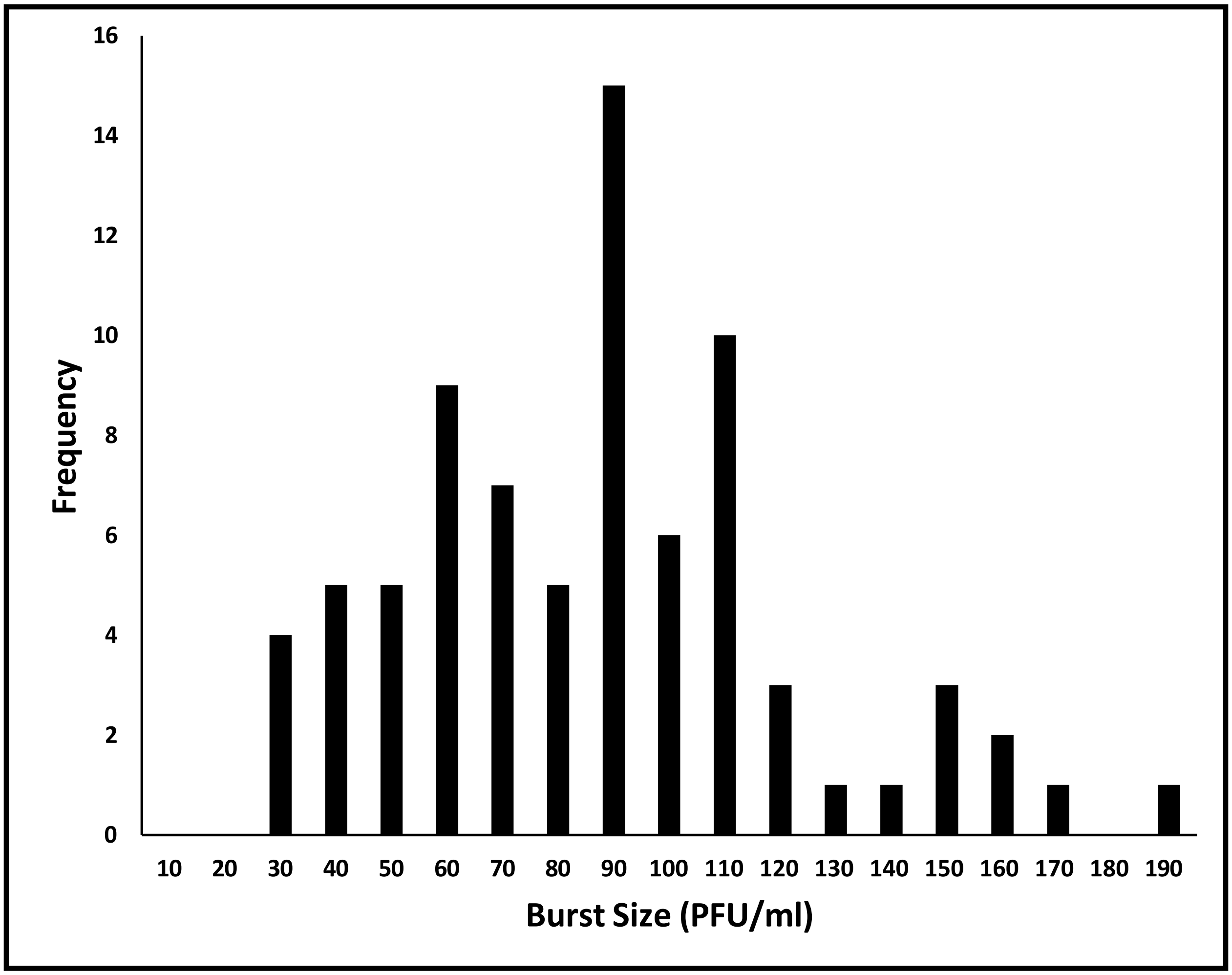

Figure S5. Burst size distribution of wild type phage λ. Following thermal induction, FACS ARIA was used to sort single FITC-labeled lysogens into wells of a 96-well plate. Cell detection was tuned to a very narrow expression profile to ensure homogeneity in cell size. Phages released from lysed cells were enumerated using plaque assays.

Burst size noise in chemically lysed cells and a naturally lysing holin mutant

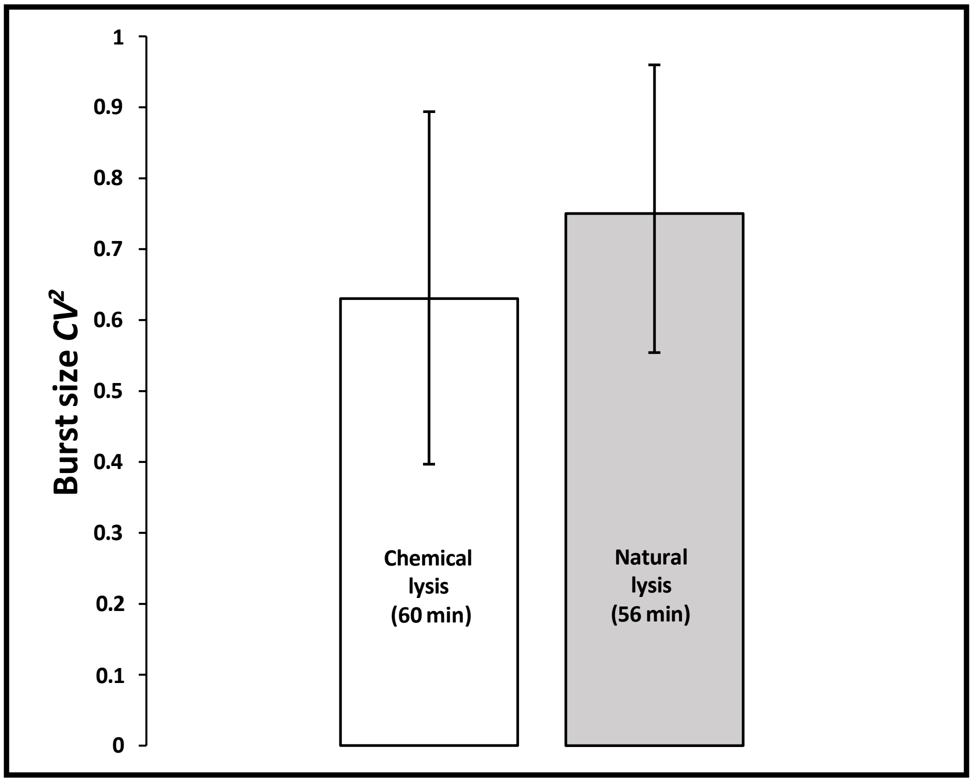

Figure S6. Chemically and naturally lysed cells show a similar cell-to-cell variation (noise) in burst size (CV^2). An induced culture of *E. coli* lysogen with lysis-deficient λ phage was chemically lysed (n ≈ 100) at 60 min to estimate the noise in burst size as quantified using the coefficient of variation squared (CV^2). This was not significantly different from the noise estimated using lysogenic cells harboring a mutant holin with a mean lysis time of 56 min (Asymptotic test, $p = 0.53$; Modified signed-likelihood ratio test, $p = 0.52$). Error bars, 95% CIs after bootstrapping with 1,000 replicates.

Burst size noise calculated after removing outliers

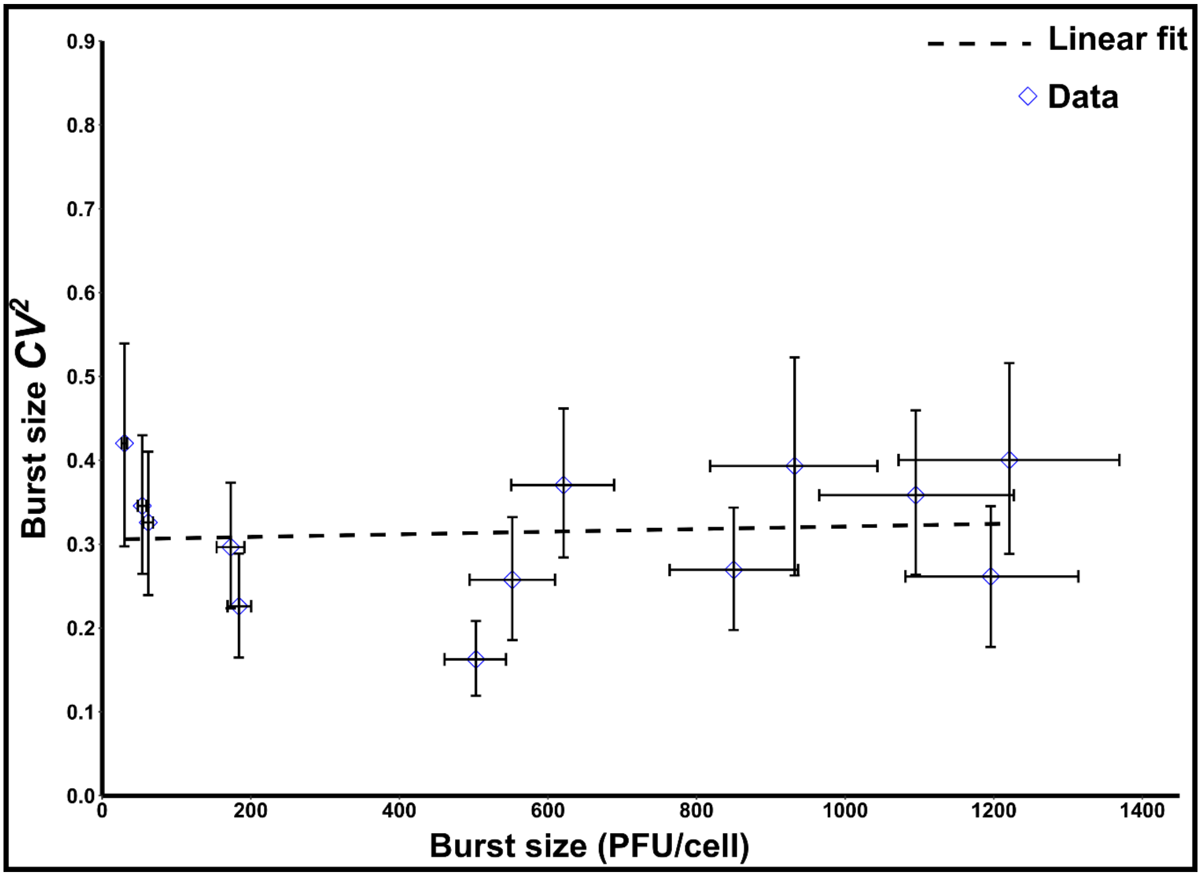

Fig. S7. After removing outliers, cell-to-cell variation (noise) in burst size remains constant with increasing mean burst size. Noise (CV^2) in burst size is shown plotted against mean burst sizes estimated for a range of lysis times. Each point represents the mean burst size estimated from ≈100 cells. The dotted line is a linear fit of the data. Error bars, 95% CIs after bootstrapping (1,000 replicates).

Supplementary Table

Time of chemical lysis (min)	Observed Mean burst size	Observed noise	Skewness	Remove outliers	Skewness after removing outliers	Mean burst size after removing outliers	% decrease in Burst Size	Original sample size (N)	N after removal of outliers
40	34	0.483266	0.554436	>68	0.192027	30	11.76471	108	99
50	62	0.489322	1.259905	>124	0.377643	54	12.90323	127	118
60	77	0.630148	2.006758	>154	0.323685	62	19.48052	112	101
80	200	0.392724	0.88186	>400	0.494868	173	13.5	101	92
90	210	0.33057	0.97746	>420	0.180112	184	12.38095	116	106
100	548	0.260765	1.518898	>1096	0.536258	503	8.211679	94	89
120	621	0.367417	1.103278	>1242	0.299421	552	11.11111	102	95
150	783	0.562369	1.13283	>1566	0.487086	621	20.68966	126	112
180	1012	0.473746	1.595238	>2024	0.499778	850	16.00791	114	104
210	1059	0.483913	0.959809	>2118	0.67964	932	11.99245	115	107
240	1412	0.421893	1.141583	>2824	0.070844	1196	15.29745	112	102
300	1273	0.445773	0.774655	>2546	0.368471	1095	13.98272	98	89
360	1445	0.490105	0.788223	>2890	0.323151	1221	15.50173	109	98

References

1. Dennehy, J. J. and Wang, I.-N. (2011) 'Factors influencing lysis time stochasticity in bacteriophage λ.', *BMC microbiology*. BioMed Central, 11, p. 174. doi: 10.1186/1471-2180-11-174.
2. Schneider, C. A., Rasband, W. S. and Eliceiri, K. W. (2012) 'NIH Image to ImageJ: 25 years of image analysis', *Nature Methods 2012 9:7*. Nature Publishing Group, 9(7), pp. 671–675. doi: 10.1038/nmeth.2089.
